# An Entropy-Based Measure of Complexity: An Application in Lung-Damage

**DOI:** 10.3390/e24081119

**Published:** 2022-08-14

**Authors:** Pilar Ortiz-Vilchis, Aldo Ramirez-Arellano

**Affiliations:** Escuela Superior de Medicina, Instituto Politécnico Nacional, Mexico City C.P. 11340, Mexico

**Keywords:** entropy, complexity measure, d-summable information dimension, lung-damage, COVID-19

## Abstract

The computed tomography (CT) chest is a tool for diagnostic tests and the early evaluation of lung infections, pulmonary interstitial damage, and complications caused by common pneumonia and COVID-19. Additionally, computer-aided diagnostic systems and methods based on entropy, fractality, and deep learning have been implemented to analyse lung CT images. This article aims to introduce an Entropy-based Measure of Complexity (EMC). In addition, derived from EMC, a Lung Damage Measure (LDM) is introduced to show a medical application. CT scans of 486 healthy subjects, 263 diagnosed with COVID-19, and 329 with pneumonia were analysed using the LDM. The statistical analysis shows a significant difference in LDM between healthy subjects and those suffering from COVID-19 and common pneumonia. The LDM of common pneumonia was the highest, followed by COVID-19 and healthy subjects. Furthermore, LDM increased as much as clinical classification and CO-RADS scores. Thus, LDM is a measure that could be used to determine or confirm the scored severity. On the other hand, the d-summable information model best fits the information obtained by the covering of the CT; thus, it can be the cornerstone for formulating a fractional LDM.

## 1. Introduction

The imaging studies X-ray and Computed Tomography (CT) are diagnostic tests for early detection of injuries, internal bleeding, diseases, cancer, and infections (pneumonia and COVID-19) in specific areas. CT is a tool for evaluating the evolution of lung infections and pulmonary interstitial damages, complications caused by common pneumonia, cancer, and COVID-19 [1,2,3,4]. The clinical scoring systems have been proposed as a guide to assess and report image findings. They are usually semi-quantitative or quantitative and do not demand significant additional resources [5,6,7,8,9]. However, medical expertise and clinic tests are essential to apply them. Furthermore, many Computer-Aided Diagnostic (CAD) systems have been implemented to improve diagnosing and monitoring the lung disease progression [10,11,12,13,14,15,16,17]. Several methods based on entropy [18,19,20,21] and fractality [22,23,24,25,26,27,28] have been proposed to analyse airway branching complexity and pulmonary parenchyma on lung CT images.

Machine and deep learning are powerful tools for detecting, diagnosing, and predicting pulmonary diseases such as COVID-19. The reader is referred to [29,30,31] for a profound discussion on the machine and deep learning CT analysis. However, these approaches need many high-quality images to be accurate in the diagnosis, which is an open issue in this field [32]. The resulting models are difficult to be interpreted by humans. In addition, a measure that quantifies the complexity, or even the severity of the damage caused by the infection is rather difficult to be extracted from them [33]. Thus, a measure of complexity of the space-filling relied on entropy has not been proposed, to the authors’ best knowledge. Furthermore, no investigations have been conducted on developing a measure of the complexity of lung damage by analysing CT images, which is the second goal of this research. The cornerstone of the two proposed measures is quantifying the information produced by how fast the space is filling by falling in [0, 1], where one means the highest complexity value.

After this introduction the related works and preliminaries are presented, followed by the Entropy-based Measure of Complexity (EMC) and Lung Damage Measure (LDM), a particular case of the first. Next, the methodology is described, and an application is presented. Finally, the discussion and conclusion are given.

## 2. Related Works

### 2.1. Entropy and Complex Approaches

Entropy measures the unpredictability that specifies a system’s degree of disorder and complexity. Entropy is the cornerstone to enhance chest X-ray pneumonia images [18], select the relevant features to train an Artificial Neural Network (ANN) to recognize pneumonia [19] and lung nodules in CT images [20]. Similarly, Tsallis–Havrda–Charvat and Shannon entropies are a loss function in a deep neural network to identify lung cancer based on CT images and clinical data [21].

Fractal analysis has been applied to medical imaging. The fractal dimension is the omnipresent measure in this analysis. It was computed on a pulmonary arterial network extracted from X-ray images to show its fractal pattern [22]. Similarly, the fractal dimension of the lung CT images was associated with morbidity caused by chronic obstructive pulmonary disease [23,24] and with the stage of lung cancer [27].

Multifractal analysis of lung CT images of patients with COVID-19 shows that the capacity dimension is correlated with the progression and reduction of lung infection [28]. The use of entropy (as a measurement of complexity) in fractal analysis raises the information dimension measure. This measure can differentiate among several lung affections [25,26].

The previous works show that the fractal and capacity dimensions are associated with the repercussion of pulmonary illnesses such as COVID-19. These repercussions include mortality, the extension and the complexity of lung damage. Furthermore, the fractal and information dimensions lack standardization and independence of the scale. It means that these measures are influenced by the size of CT and could take a wide range of values. Thus, it is rather difficult to compare the results in different contexts.

### 2.2. Artificial Intelligence

Machine-learning and deep-learning are areas of artificial intelligence that employ multi-layered artificial neural network models. They have important applications in clinical practice, focusing on modelling, classification and diagnosis without human interaction.

Artificial intelligence algorithms have been used for disease modelling, classification, and characterisation [34,35,36]. They pursue the discovery of hidden associations in the data [37], identifying key clinical variables, making predictions to support decisions [38], reducing laboratory tests [35,39] and processing images to diagnose [34,40,41,42,43]. The ultrasound image of the carotid atherosclerotic plaque tissue is analysed by a 3-D deep convolution neural network [34] to identify the tissue rupture that can be the precursor of a stroke. Similarly, the author of [43] proposed a convolutional neural network for the automatic segmentation of chest X-ray to diagnose cardiomegaly. Furthermore, the electrocardiogram analysis by the convolutional neural network has been employed to identify five types of arrhythmic heartbeats [42] and guide the screening process for subcutaneous implantable cardioverter-defibrillators [44]. The convolutional neural network has analysed CT to identify cerebral infarction [40] and pulmonary fibrosis [41].

Several convolutional neural networks have also been used to identify infection of COVID-19 in X-ray and CT images for quick and accurate diagnosis [45,46,47,48,49,50]. ResNet-101 and Xception could characterize and diagnose COVID-19 infections with high sensitivity, specificity, and accuracy between 80–99% [45,49,50] compared to the radiologist’s performance with a sensitivity, specificity, and accuracy of less than 90% [45]. New algorithms have been designed to classify and detect COVID-19. MODE convolutional neural networks-based on classifying COVID-19-infected patients with lesser false-negative and false-positive values [46]. FGCNet fuse a graph convolutional network and a convolutional neural network to detect COVID-19 from chest CT images. Another tailored deep convolutional neural network, COVIDNet-CT, was designed to detect COVID-19 in CT images with a high test accuracy (99.1%) and low architectural and computational complexity [51].

The approaches based on deep neural networks and machine learning techniques have shown high accuracy rates in classifying and diagnosing COVID-19 disease using CT images. However, a measure that quantifies the complexity and the severity of the damage caused by lung illnesses is difficult to be extracted from them. Thus, indirect measurements are computed instead based on the lesions identified by the artificial neural networks. For example, the change in the volume of the lesions from CT at two different times [1] is compared to measure the progression of the illness. Similarly, the z-score between the average volume of lung opacities in healthy subjects and the opacities caused by lung lesions of COVID-19 are used to classify the illness as mild, moderate and severe [13]. Both measures quantify the volume of the lesions but not their complexity.

## 3. Preliminaries

### 3.1. Fractal and Information Dimensions

A fractal dimension quantifies the complexity of a pattern or set as a ratio of the change in detail to the change in scale. Mandelbrot [52] defines a fractal as a subset of the Euclidean space with a fractal dimension that strictly exceeds its topological dimension. The Hausdorff dimension of bounded and closed sets can be obtained by counting the finite coverings by closed boxes, which satisfies the condition that the intersection of the interiors of any pair of boxes is empty. The so-called box-counting dimension is more appropriate than the Hausdorff dimension for measuring a given set’s fractality (e.g., see [53]. The box-counting dimension of a compact set $E\subset R^{n}$ is defined as follows:

(1) $$d_{b}=\lim_{\varepsilon \to 0}\frac{\log N(\varepsilon )}{-\log\varepsilon },$$

where N(ε) stands for the minimal number of boxes of diameter at most ε needed to cover E, and the size of the box ε has a range of values that produces the set of points ε vs. N(ε).

**Figure 1:**
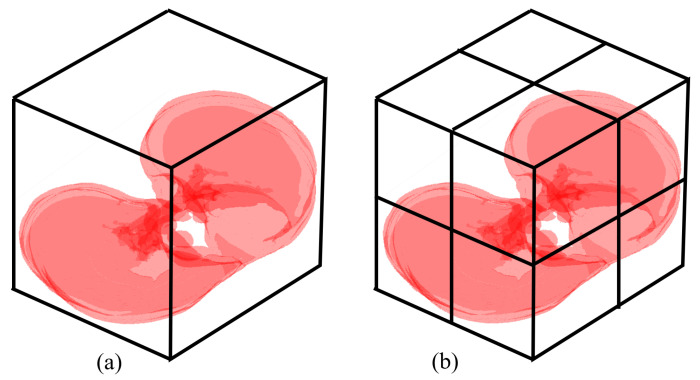
The covering of the CT by cubes of size (**a**) *s* and (**b**) s/2.

**Figure 2:**
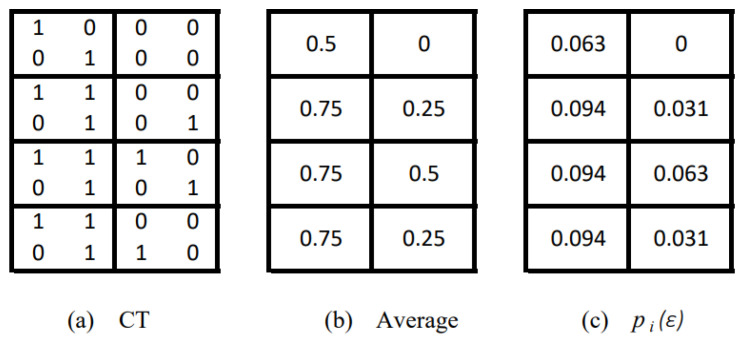
Computation of the probabilities of each box of size ε=s/2.

The information dimension was introduced in [54] as follows:

(2) $$d_{I}=-\lim_{\varepsilon \to 0}\frac{I_{c}\left(\varepsilon \right)}{\log\varepsilon }=\lim_{\varepsilon \to 0}\frac{\sum_{i=1}^{N}p_{i}\left(\varepsilon \right)\log p_{i}\left(\varepsilon \right)}{\log\varepsilon },$$

where $p_{i}\left(\varepsilon \right)$ is the probability of a symbol given a box of diameter ε, and *N* is the number of boxes covering the set. The term box is general known, but for CT it should be a cube. If all events are uniformly distributed, i.e., $p_{i}=1/w$, the entropy I(ε) is maximum [55]. For example, the CT of Figure 1a is covered by a cube of size ε=s, where *s* is the width and height of the CT. For simplicity, we assume that the CT has *s* slices. Figure 1b shows the covering by $2^{3}$ cubes of size s/2. Now, the probability $p_{i}\left(\varepsilon \right)$ is calculated by first normalising all pixels in each cube by dividing its value by the max intensity [56]; in our example, each pixel has a value of 0 or 255, and each cube has four pixels. Thus, this process results in the table in Figure 2a. Then the mean of each box is computing, see Figure 2b. Finally, the mean is divided by the total number of boxes to obtain $p_{i}\left(\varepsilon \right)$.

By (2), we can assert that

(3) $$I_{c}\left(\varepsilon \right)∼-d_{I}\log\varepsilon +\beta ,$$

for some constant β, where ε is the size of the boxes covering the set.

### 3.2. D-Summable Information Dimension

A bounded and closed set is said to be d-summable if and only if the improper integral converges.

(4) $$\int_{0}^{1}N\left(\varepsilon \right)\;\varepsilon^{d-1}\;d\varepsilon ,$$

N(ε) is the number of boxes of diameter ε covering E, and *d* is the fractal dimension. The reader is referred to a detailed discussion of d-summable sets [57].

The d-summable dimension [58] is defined as follows:

(5) $$\frac{N(\varepsilon )}{\varepsilon^{1-\nu }}∼C\varepsilon^{-d},$$

where *d* is the d-summable dimension, and *N* is the number of boxes of size ε needed to cover the set.

From (4), (5) and (2), we deduce that

(6) $$d_{dI}=\lim_{\varepsilon \to 0}\frac{\frac{\sum_{i=1}^{N}p_{i}\left(\varepsilon \right)\log p_{i}\left(\varepsilon \right)}{\varepsilon^{1-\nu }}}{\log\varepsilon },$$

where ε is the size of the boxes, and $p_{i}\left(\varepsilon \right)$ is the probability of a given box. The information dimension is obtained when ν→1. For more details on the definition of the d-summable information dimension, the reader is referred to [58].

The information (2) and d-summable information (6) dimensions have an indeterminacy, because for ε=1, the denominators of both equations are zero. This indeterminacy is solved in [59], which reformulates the information dimension by restricting the box size to [2,Δ−1], where Δ is the maximum size needed to cover the set with one box fully.

Similarly, for some constant β, we see that

(7) $$dI\left(\varepsilon \right)∼\left(-d_{dI}\log\varepsilon +\beta \right)\varepsilon^{1-\nu },$$

where $d_{dI}$ is the d-summable information dimension, and ε is the size of the boxes necessary to cover the set.

### 3.3. Entropy-Based Measure of Complexity

The entropy-based measure of complexity is defined as:

(8) $$EMC=1-\int_{a}^{b}\frac{I(\varepsilon )\;d\varepsilon }{\frac{\left(b-a\right)(I_{max}\left(a\right)-I_{max}\left(b\right))}{2}+I_{max}\left(b\right)\left(b-a\right)},$$

where I(ε) is the information function such as the classical information (3) or d-summable (7). The physical meaning of EMC is the quantification of the information of how fast the space is filled by changing the scale. Note that EMC is a normalised measure where one means that the information speed for filling the space is the highest. The lung damage measure can be derived from (8), considering the following. Since the CT are stored as binary objects, the size of each cube to cover the CT is $\varepsilon =[a=2,4,\ldots ,b=2^{log_{2}\left(s\right)-1}]$, where *s* is the size of the CT p.e. 512 pixels. $I_{max}\left(\varepsilon \right)$ is obtained when $p_{i}\left(\varepsilon \right)=1/w$, where *w* is the number of cubes of size ε to cover the CT. Moreover, $I_{max}\left(\varepsilon \right)=\log{\left(\frac{s}{\varepsilon }\right)}^{3}$. The lung damage measure is defined as:

(9) $$LDM=1-\int_{a}^{b}\frac{I(\varepsilon )\;d\varepsilon }{\frac{\left(b-a\right)\left(\log{\left(\frac{s}{a}\right)}^{3}-\log{\left(\frac{s}{b}\right)}^{3}\right)}{2}+\log{\left(\frac{s}{b}\right)}^{3}\left(b-a\right)}.$$

In practise, $\int_{a}^{b}I\left(\varepsilon \right)\;d\varepsilon$ can be approximated by computing $I{\left(\varepsilon \right)}_{c}=-\sum_{i=1}^{N}p_{i}\left(\varepsilon \right)\log p_{i}\left(\varepsilon \right)$; then it can be integrated numerically.

**Figure 3:**
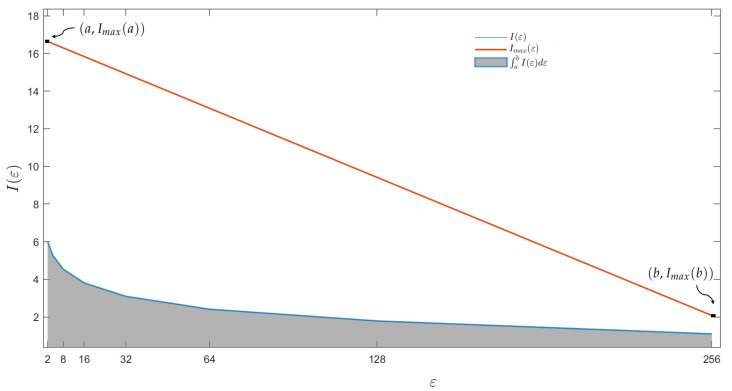
The geometrical interpretation of the lung damage measure.

The geometrical interpretation of LDM is shown in Figure 3. The blue line is I(ε) for [a,…,b] obtained by the CT’s box-covering. Thus, the gray area is $\int_{a}^{b}I\left(\varepsilon \right)\;d\varepsilon$. The orange line between the points $\left(a,I_{max}\left(a\right)\right)$ and $\left(b,I_{max}\left(b\right)\right)$ means the maximum entropy $I_{max}\left(\varepsilon \right)$ for each ε. Thus, $I_{max}\left(a\right)=\log{\left(\frac{s}{a}\right)}^{3}$ and $I_{max}\left(b\right)=\log{\left(\frac{s}{b}\right)}^{3}$. The divisor of (9) is the area under max entropy $I_{max}\left(\varepsilon \right)$ (orange line) plus the area of the rectangle $\left(b-a\right)I_{max}\left(b\right)$. The second term of (9) means the area of the CT divided by the area of the max entropy that can be obtained by an object of the same size. I(ε) (blue line) is expected to decay faster for a complex object. Thus, $\int_{a}^{b}I\left(\varepsilon \right)\;d\varepsilon \to 0$ and LDM→1; on the less complex object occurs the opposite.

Now, we are ready to give an interpretation of LDM in the context of medical imaging. A healthy lung is mostly an empty space (the CT is mostly black), so the information on how fast the space is filled should be low (little information is contained no matter the size of the cubes to cover the lungs). On the contrary, lung lesions (lung volume loss, septal thickening, halo sign, bronchial dilatation, centrilobular nodules, ground-glass opacities, consolidations, crazy paving) appear on a CT as a white region. The entropy for a given scale I(ε) quantify the lesion extension (space-filling as ones in the boxes) see Figure 2a, and how complex they are (the entropy computed using the $p_{i}\left(\varepsilon \right)$ shown in Figure 2c. Integrating this information $\int_{a}^{b}I\left(\varepsilon \right)\;d\varepsilon$, we have a global measure of both. In summary, the LDM measures the extension and complexity of the lesions.

## 4. Method

**Figure 4:**
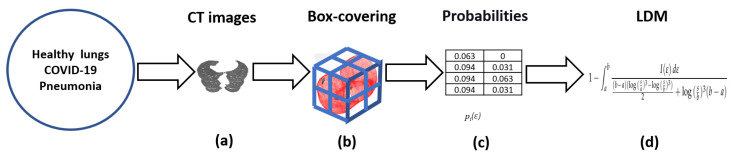
The steps to compute the LDM.

The CT of subjects with healthy lungs (486), COVID-19 confirmed diagnosis (263) and those with common pneumonia (329) included in this study were gathered from [60]. In addition, COVID-19 (49) with clinical description data were collected from Radiopaedia [61]. The search criteria were: “cases”, “breast system”, “CT study modality”, “COVID-19”, and “adult”. The selected CT were those in which the slices covered the lung region completely. The lungs were selected as the Region Of Interest (ROI) using a mask. The image of each slice was converted to a matrix then they were piled to build a CT cube of 512 by 512 by the number of slices, see Figure 4a. Once the CT cube was built, the box-covering algorithm was employed to compute the number of cubes *N*, of size ε, to cover the regions filled up in the CT, see Figure 4b. Finally, the $p_{i}\left(\varepsilon \right)$ is obtained from each cube, as was explained, see Figure 4c. Finally, the LDM is computed, see Figure 4d. The ROI selection was performed automatically using ad-hoc software. Four expert radiologists validated the ROI.

The LDM computed on the CT of healthy, COVID-19, and common pneumonia were analysed using a Kruskal–Wallis test to show the effect of these diseases. Since the LDM was neither normally distributed nor homoscedastic the Kolmogorov–Smirnov and Levene tests were employed to verify the non-normality and non-homoscedasticity, respectively. A statistical analysis focused on Pearson’s correlation coefficient (r) between the clinical classification of The National Commission of Chinese Health, the COVID-19 Reporting and Data System (CO-RADS), and LDM was carried out using CT of COVID-19 (49) collected from Radiopaedia [61]. According to [62], the clinical classification distinguishes four levels of gravity (mild, moderate, severe and critical cases) based on clinical manifestations and imaging findings. CO-RADS is a scoring system (0–6 points) based on stratifying the suspicion of pulmonary involvement in COVID-19 using the chest CT findings to make decisions in clinical practice. The score increases according to suspiction from insufficient evidence (category 0), very low to very high (categories 1–5), and confirmed by polymerase chain reaction (PCR) (category 6) [63]. The individual effect of clinical classification and CO-RADS on LDM was tested using a one-way ANOVA and Bonferroni post hoc. These statistical analyses were performed using SPSS Statistics 25; meanwhile, the computation of LDM was performed in Matlab R2022a.

The Akaike Information Criterion (AIC) [64] was employed to select from (3) and (7) the best model that describes the information obtained from each CT. The AIC balances the goodness-of-fit and the complexity of the model (number of parameters) in a single measure to avoid over-fitting. The parameters of (3) and (7) were approximated by non-linear regression [65].

## 5. Applications

The number of CT, the average number of slices and LDM computed on healthy, COVID-19, and common pneumonia are shown in Table 1. The Kolmogorov–Smirnov and Levene test on LDM show that it is not normally distributed nor homoscedastic, respectively. The Kruskal–Wallis test showed a significant difference in the illnesses on LDM, H(2)=428.349, p<0.0001. The pairwise comparisons (Mann–Whitney test) using the Bonferroni adjustment [66] showed a significant difference of LDM between healthy subjects, and those suffered from COVID-19 $U(N_{h}=486,N_{c}=263)=32,157$, z=−11.234, p<0.001 and common pneumonia $U(N_{h}=486,N_{p}=329)=15,362$, z=−19.587, p<0.001. Additionally, the COVID-19 and common pneumonia comparison showed a significant difference of LDM $U(N_{c}=263,N_{p}=329)=$ 22,908, z=−9.844, p<0.001.

**Figure 5:**
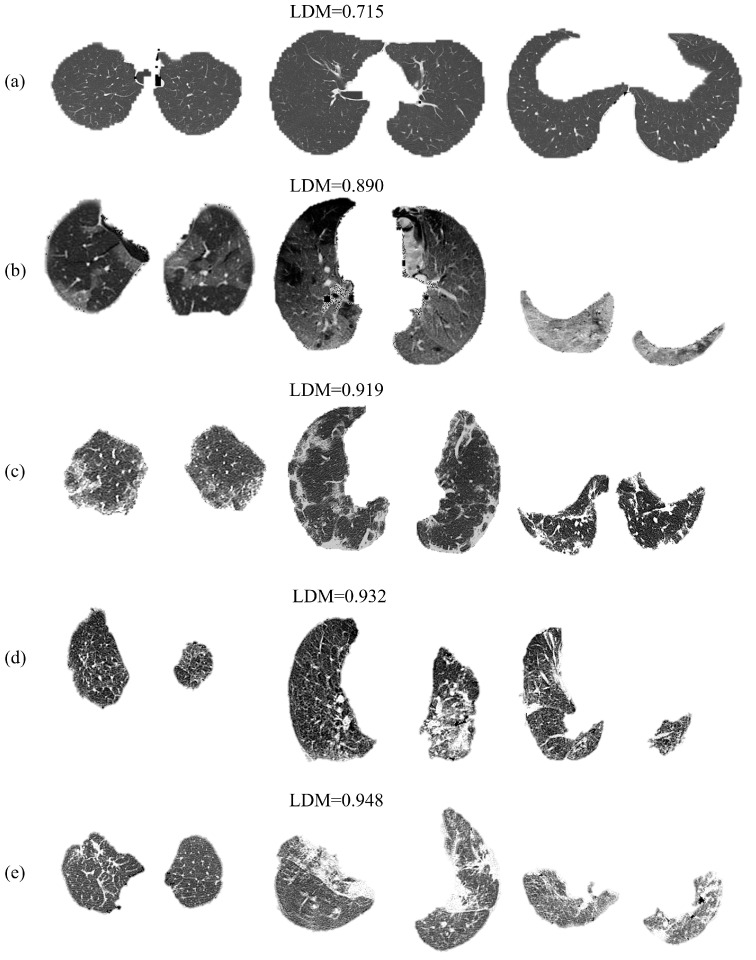
Slice lungs of healthy (**a**), COVID-19 (**b**,**c**), and pneumonia (**d**,**e**).

Figure 5 show slice lungs of healthy, COVID-19, and common pneumonia. The LDM of a healthy subject (a) is the lowest, followed by COVID-19 (b,c), where the complexity of the lesions is higher. The COVID-19 and common pneumonia image findings are paved, bronchus distortion, bronchiectasis and consolidation in bilateral involvement, see Figure 5b–e. However, the ground glass opacification is evident only in COVID-19 slices. Furthermore, atelectasis and peribronchovascular thickening are only presented in common pneumonia examples, Figure 5d,e. The LDM quantify the complexity of these findings by the roughness and fractured patterns in those lesions. For example, the lesions in Figure 5e present more sharpened edges than those in Figure 5c.

**Figure 6:**
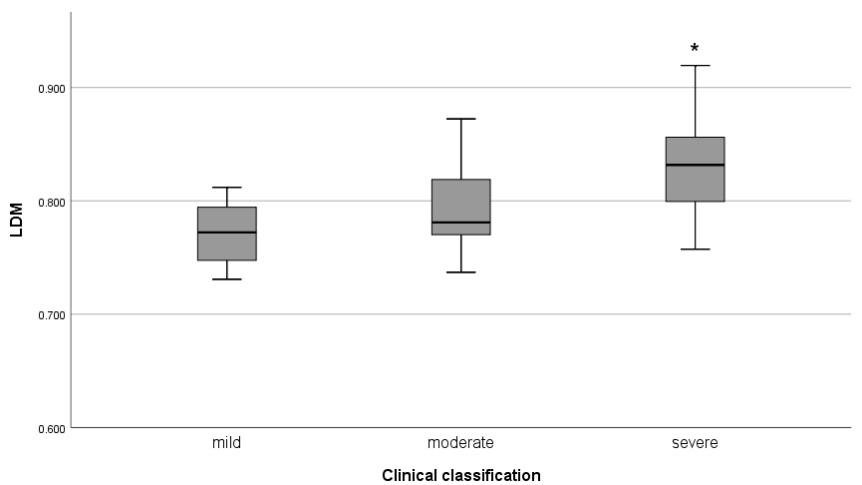
The LDM for clinical classification score. * Means that LDM of mild and severe were statistically different.

The CT of subjects suffering from COVID-19 (n = 49, gathered from Radiopaedia) was scored using the clinical classification and CO-RADS. Kolmogorov–Smirnov and Levene test on LDM (μ=0.810, σ=0.048) shows that it is normally distributed and homoscedastic by both scores. A Pearson’s coefficient was calculated to assess the correlation between LDM and clinical classification (r(46)=0.49, p=0.01), and LDM and CO-RADS (r(46)=0.47, p=0.01). In addition, the ANOVA show an effect of clinical classification on LDM (F(2,45)=7.377, p=0.002). A statistical difference was found between level mild, (μ=0.779, σ=0.043) and severe (μ=0.831, σ=0.042) by Bonferroni post hoc. Although, no significant difference was found between level mild (μ=0.779, σ=0.043) and moderate, (μ=0.794, σ=0.043) and between level moderate, (μ=0.794, σ=0.043) and severe (μ=0.831, σ=0.042) the LDM increases as clinical classification, see Figure 6. The critical level was not identified because no extensive consolidations or white lung were notorious in the CT.

**Figure 7:**
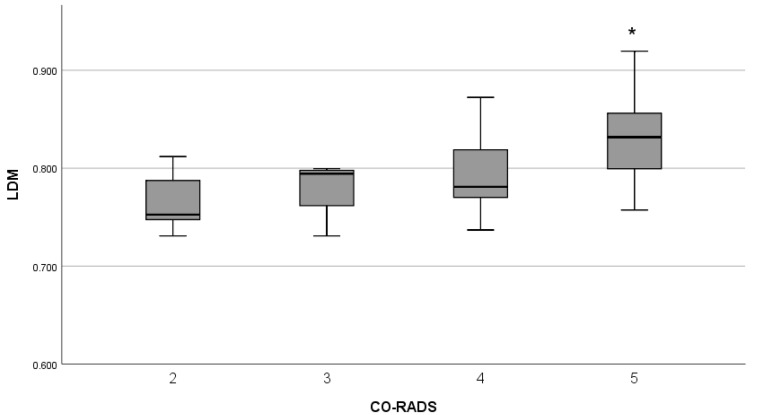
The LDM for CO-RADS score. * Means that LDM of levels two and five were statistically different.

The ANOVA shows that the CO-RADS score affects LDM (F(3,44)=4.810, p=0.006). A statistical difference was found only between level two (μ=0.779, σ=0.049) and five (μ=0.8310, σ=0.042) by Bonferroni post hoc while no significant difference was found between the remaining levels in multiple comparisons (level three μ=0.780, σ=0.033, level four μ=0.794, σ=0.043). However, LDM increased as much as the CO-RADS score, see Figure 7. Levels one and six were not considered due to not being based on tomographic findings.

The functional form of I(ε) was selected from (3) and (7) using the AIC [64]. First, the minimum value of AIC of two models is chosen $AIC_{min}$; then each $\Delta AIC_{i}=AIC_{i}-AIC_{min}$ is computed, where *i* is the candidate model; i=I for (3) and i=dI for (7). The rule of thumb is that two models fit the data equally if $\Delta AIC_{i}<2$ [67,68]. On the other hand, if ΔAIC≥2, there is sufficient statistical evidence to choose one over the other. Table 2 shows the average and standard deviation of $AIC_{i}$ and $\Delta AIC_{i}$ for information model (*I*) and d-summable information model (dI) as well as information dimension ($d_{I}$), d-summable information dimension ($d_{dI}$), and ν. The d-summable information model (7) was selected for healthy and COVID-19 CT and mostly for those lungs affected by common pneumonia. For twenty-eight out of 320 CT affected by common pneumonia, there is no difference in selecting the information model or d-summable information model since ΔAIC<2 for both models. Thus, the d-summable information model describes the complexity of the analysed CT. Note that the $d_{I}$ and $d_{dI}$ are higher for healthy lungs and decrease for COVID-19 and common pneumonia.

## 6. Discussion and Conclusions

This study aimed at introducing a measure of the information produced by how fast the space is filled by changing the scale named EMC. A particular case of EMC called LDM was applied on CT images to differentiate the lesions caused by COVID-19 and common pneumonia. The results showed that the LDM statistically differs between healthy, common pneumonia, and COVID-19 lungs. This means that the complexity of healthy lungs is the lowest; meanwhile the complexity increased for COVID-19 and common pneumonia. The fractal dimension has been used to analyse pulmonary vessel and airway branching patterns and pneumonia lesion detection on X-ray and CT images [23], but not to quantify the complexity of the lesions. Moreover, the LDM could be the cornerstone of automatic-computational diagnostic systems. The complexity of the lungs affected by COVID-19 and common pneumonia is higher than healthy lungs; thus, these findings align with previous studies performed in both X-ray and CT images [25,28].

The image findings and clinical scoring systems help diagnose and classify patients’ health status. Our findings show that LDM increases as much as the scored severity of patients’ illnesses. Hence LDM is a measure that could be used to determine or confirm the scored severity. In addition, the d-summable information model best fits the information $I_{c}\left(\varepsilon \right)$ obtained by the covering of the CT; thus, it paves the way to formulate a fractional LDM based on this model. The EMC can be applied to complex networks, or individual images such as X-rays. Furthermore, the LDM (designed for three dimensional objects) is not restricted to the lungs. It could be applied to other organs such as the liver or kidneys.

## Figures and Tables

**Table 1:** Chest tomography features. Results are expressed as mean (μ) and standard deviation (σ).

Disease	CT Number	Slice Number	LDM
Healthy lungs	486	90.300 (8.703)	0.779 (0.031)
COVID-19	263	65.027 (16.084)	0.814 (0.042)
Common pneumonia	329	86.611 (14.262)	0.852 (0.045)

**Table 2:** The AIC and ΔAIC from information model (*I*) and d-summable information model (dI). Results are expressed as mean (μ) and standard deviation (σ).

Disease	${\mathrm{AIC}}_{I}$	${\mathrm{AIC}}_{\mathrm{dI}}$	$\Delta {\mathrm{AIC}}_{I}$	$\Delta {\mathrm{AIC}}_{\mathrm{dI}}$	$d_{I}$	$d_{\mathrm{dI}}$	ν
Healthy lungs	−24.182 (3.255)	−42.927 (1.760)	15.14 (2.782)	0 (0)	1.016 (0.164)	1.007 (0.165)	1.019 (0.004)
COVID-19	−23.085 (2.53)	−42.273 (3.1384)	15.58 (2.601)	0 (0)	0.838 (0.211)	0.826 (0.213)	1.027 (0.009)
Common pneumonia	−30.146 (9.990)	−46.358 (6.035)	12.986 (5.601)	0.08 (0.352)	0.637 (0.231)	0.628 (0.230)	1.024 (0.001)

## Data Availability

The data that support the findings of this study are available from the corresponding author upon reasonable request.

## Abbreviations

The following abbreviations are used in this manuscript:

AIC	Akaike Information Criterion
ANOVA	Analysis of Variance
CAD	Computer-aided Diagnostic
CO-RADS	Classification of the NCCH, the COVID-19 Reporting and Data System
COVID-19	Coronavirus Disease 2019
CT	Computed Tomography
EMC	Entropy-based Measure of Complexity
LDM	Lung Damage Measure
PCR	Polymerase Chain Reaction
ROI	Region Of Interest
